# A Case of Protracted Febrile Myalgia Syndrome with Atypical Course and Severe Asymmetric Loss of Muscle Strength

**DOI:** 10.31138/mjr.300723.aco

**Published:** 2023-07-30

**Authors:** Rabia Deniz, Aybüke Mandacı, Ilayda Gerdan, Duygu Sevinç Özgür, Bilgin Karaalioğlu, Gamze Akkuzu, Fatih Yıldırım, Cemal Bes

**Affiliations:** 1 Department of Rheumatology, University of Health Sciences Başakşehir Çam and Sakura City Hospital, Istanbul, Turkey,; 2 Department of Internal Medicine, University of Health Sciences Başakşehir Çam and Sakura City Hospital, Istanbul, Turkey,; 3 Department of Physical Medicine and Rehabilitation, University of Health Sciences Istanbul Physical Medicine and Rehabilitation Hospital, Istanbul, Turkey

**Keywords:** anakinra, familial Mediterranean fever, protracted febrile myalgia, P369S mutation

## Abstract

Protracted febrile myalgia syndrome (PFMS) is a rare form of familial Mediterranean fever (FMF) characterised by prolonged myalgia. The duration of PFMS is much longer than a typical 2–5-day attack familial Mediterranean fever and lasts for 2-6 weeks until they treated with corticosteroids. Colchicine is not effective for control of PFMS’s attacks. The attacks typically resolve with corticosteroid and/or IL-1 receptor blockers. Herein, we present a young adult without typical familial Mediterranean fever clinical features but presenting with atypical course and severe asymmetric muscle strength loss. Thigh magnetic resonance imaging confirmed inflammation and oedema and muscle biopsy showed no pathological findings. Electromyography revealed myopathic findings during attack-period, despite normal results in attack-free study. The patient was treated successfully with anakinra and remarkable rapid recovery in both muscular findings and acute phase reactants were observed. PFMS should be considered even in the absence of apparent FMF attack pattern and in the presence of unexpected severe muscle weakness, especially in areas endemic for FMF and long-lasting myalgia attacks.

## INTRODUCTION

Familial Mediterranean Fever (FMF) is an autosomal recessive inherited disorder caused by mutations of the MEFV gene encoding pyrin protein, a regulator of IL-1 related inflammatory pathway. It manifests as self-limiting, recurrent febrile inflammatory attacks of serosal and synovial membranes. Additionally, arthritis, arthralgia, myalgia and erysipelas-like erythema, and more uncommonly protracted febrile myalgia syndrome (PFMS) are observed.^1–4^ Here, we presented an atypical case of PFMS with severe asymmetrical loss of muscle strength, afebrile clinic, and a rarer mutation.

## CASE PRESENTATION

A 25-year-old Caucasian male patient presented with intermittent recurrent myalgia, especially in the gastrocnemius and thigh region, for the last 5 years. He was born in Antalya, Turkey and consanguinity was described between parents. There was no known chronic disease or drug use, and similar family history. In many previous emergency service visits with similar pain attacks, C-reactive protein (CRP) was between 30 and 100 mg/dl, erythrocyte sedimentation rate (ESR) between 50–60 mm/h, procalcitonin and creatinine kinase (CK) were found to be normal. He stated that the pain lasted for 2–4 weeks and was localized in all four extremities, most prominently in the proximal lower limbs. The last six months, attacks have become more frequent, and severity of muscle pain increased as well. Fever, abdominal, flank or chest pain suggesting serositis was not accompanying this complaint. He did not have oral aphthae, genital ulcer, subileus, or diarrhoea, arthritis, psoriasis, but he stated that he had intermittent redness and mild to severe arthralgia around the bilateral malleolus. Anti-nuclear antibody (ANA), extractable nuclear antigen (ENA), anti-cyclic citrullinated peptide (anti-CCP), rheumatoid factor (RF), complement level, viral serology, and Brucella serology were all negative. Echocardiography was unremarkable except for stage 1 left ventricular diastolic dysfunction. Eye and ear nose throat examination performed for systemic involvement was found to be normal. No lymphadenopathy was detected in the abdomen, cervical, axillary, and inguinal regions in the ultrasonography examination performed in terms of foci suggestive of lymphoproliferative disease and acute phase elevation. Bilateral lower extremity arterial and venous Doppler ultrasonography was normal. At the patient’s admission, muscle strength was 5/5 in all four extremities, and laboratory findings were given in **Table 1**. Cranial, cervical, thoracic, lumbar, and sacroiliac magnetic resonance imaging (MRI) were normal except for non-compressing mild bulging at the level of a few vertebrae. Sensory and motor conduction examinations were found to be normal in the electromyography (EMG) performed during the attack-free period.

**Table 1.** Laboratory parameters of the patient during follow-up.

**Parameter**	**At hospitalisation**	**At attack-time**	**At the 1st week of anakinra**
**ESR (mm/h)**	87	N/A	9
**CRP (mg/dL)**	48	137	1
**Serum amyloid A mg/dl (ULN <0.5)**	80.3	N/A	N/A
**Urine erythrocyte**	1	1	1
**Urine leucocyte**	1	1	1
**uPCR**	88 mg/day	376 mg/day	125 mg/day
**Serum creatinine (mg/dL)**	0.77	0.66	0.71
**Serum creatinine kinase(U/L)**	33	26	53
**Ferritin (ng/mL)**	319	780	440
**WBC 10^6^/L**	8900	14900	5600
**Neutrophil 10^6^/L**	5900	12700	3100
**Lymphocyte 10^6^/L**	2600	1930	1900
**Haemoglobin g/dL**	14.1	12.1	13.0
**Platelets 10^9^/L**	443	323	405

When a 37.8° subfebrile fever developed suddenly and no infective focus was detected in the follow-up, significant deterioration in examination findings were observed. The patient developed severe and asymmetrical loss of muscle strength that caused him to remain immobile, in addition to exacerbated myalgia. Muscle strength was found as neck flexion 4/5, neck extension full, upper extremity proximal muscle groups 3-4/5, left dominant weakness, upper extremity left distal muscle group 3/5, lower extremity proximal group right 2/5 left 3-4/5, distal muscle groups were determined better on the right and left as 4-5/5. Myotonia and cramps were not detected. The attack-time laboratory showed in **Table 1**.

**Figure 1.**
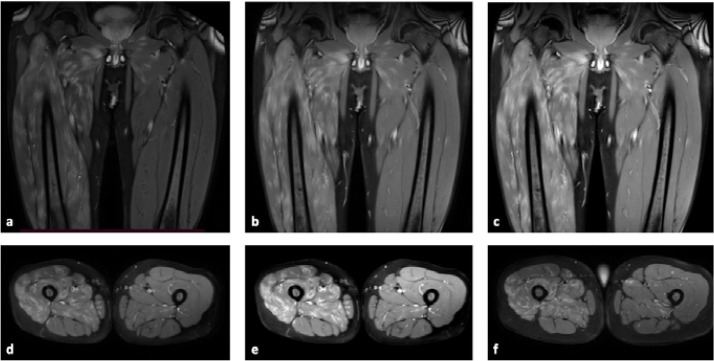
Coronal **(a)** proton density weighted (PDW) with fat saturation, **(b)** contrast enhanced T1-weighted fat saturation and **(c)** T1-weighted; and axial **(d)** proton density weighted (PDW), **(e)** contrast enhanced T1-weighted fat saturation and **(f)** T1-weighted MRI of the thigh muscles at presentation. There is remarkably increased intensity and swelling within the muscles representing severe oedema and inflammation more prominent on the right side.

On MRI of the thigh during the attack, widespread inflammation was observed in all muscle groups of the right thigh, adductor muscles on the left, and more prominent pelvic floor muscles on the bilateral right, and it was interpreted as myositis and severe oedema (**Figure 1**). Considering the current severe clinic, positron emission tomography (PET/CT) was taken during the attack and no signs of malignancy were observed. When there was a significant loss of muscle strength during the attack, repeated EMG showed an increase in needle entry activity in the right vastus medialis muscle, myogenic MUAP activity with positive sharp waves and fibrillation potentials, and an early participation pattern. Early involvement was observed with prominent myopathic MUAP activity in the left vastus medialis, right lumbar paraspinal, gluteus maximus, tibialis anterior, gastrocnemius caput medialis, flexor dig superficialis, and proximal deltoid, biceps muscles. In conclusion, fibrillation, and positive sharp wave potentials observed in the right vastus medialis muscle were interpreted as findings consistent with significant primary muscle fibre involvement in the proximal upper and lower extremities.

In the biopsy taken from the right vastus lateralis, there was no rounding of the muscle fibres and no difference in diameter, no increase in the centrally located nuclei, necrosis, myophagocytosis, endomysial and perimissial inflammatory cell infiltration, and regeneration were not observed. The glycogen, neutral lipid, NADH, SDH, COX and phosphorylase contents of the cells were normal. No increase in endomysial connective tissue was observed. Immunohistochemical examination was negative for MHC-I. No myositis or any other muscle pathology was detected.

The patient was started on 100 mg/day of anakinra subcutaneously which was given empirically after exclusion of malignancy to suppress inflammatory response. The next day, the muscle strength improved apparently, and myalgia disappeared even after first dose and found as 5/5 in all four proximal and distal extremities without any difficulty in walk and move, and the CRP decreased from 137 mg/dl to 30 mg/dl. In the first week laboratory and clinical recovery was achieved (**Table 1**). Considering prolonged febrile myalgia with atypical presentation, colchicine 1 mg/day was started to prevent AA amyloidosis in addition to anakinra and titrated to 1.5 mg/day one week later when he tolerated it well. In the MEFV gene analysis of the patient, P369S mutation was found to be homozygous for the c.1105C>T position in the exon 3 of the MEFV gene. On the last visit at the 3rd month of diagnosis, he was still under same regimen without any attack including myalgia and acute phase markers were still within normal limits.

## DISCUSSION

Myalgia is not a rare manifestation in FMF and found up to 25% of patients with three different clinical patterns.^5^ Although exercise-induced myalgia, lasting for 1–3 days, and spontaneous myalgia, lasting for 8 hours, consist of the majority of myalgia in FMF patients, approximately 11% of all cases of myalgia related with PFMS lasting for 2–6 weeks.^5,6^

The duration PFMS is much longer than a typical 2–5 days FMF attack and lasts for 2–6 weeks until they treated with corticosteroids. Colchicine itself is not usually enough both to prevent recurrence of PFMS attacks and control the attacks. Similarly, non-steroidal anti-inflammatory drugs usually remains ineffective to attack control and high dose of corticosteroids ranges from 1 mg/kg/day to pulse steroids is the mainstay of treatment leading resolution of symptoms within 5–7 days.^7^ In a few cases, especially in case of corticosteroid contraindications or resistance, IL-1 blockage with anakinra was shown to be highly effective and led to immediate attack control with excellent recovery of myalgia and acute phase reactants.^8–10^ Same observation was confirmed in our case, and anakinra exhibited excellent disease control and clinical and laboratory recovery was obtained with the first dose of anakinra.

In that case report, we presented a case of PFMS with atypical presentation in many sides. First of all, the patient had no typical FMF attack pattern with obvious fever and serositis. Atypical presentation like afebrile clinic or the initial presentation of FMF as PFMS is even more uncommon and reported just in a few cases so far.^11,12^ Our case has also denied any previous fever attack and showed subfebrile fever as 37.8 degree of body temperature at proven attack during hospitalisation. When we consider the no history of subfebrile fever, it is somehow acceptable because of increased attack frequency and severity.

Although severe myalgia and diffuse muscle tenderness on palpation are expected during the PFMS attacks, loss of muscle strength and abnormal EMG findings are not a common finding as we described in our patient and led us to exclude muscular pathologies.^8^ In literature, one case of myositis with FMF and ankylosing spondylitis overlap patient with abnormal EMG findings was described but this case had also elevated CK levels and thigh MRI confirming myositis, all differentiated it from our case.^13^

MRI is an important tool in initial evaluation of involved muscle to make differential diagnosis.^14^ Despite severe paralysing myalgia and MRI findings suggesting severe oedema and inflammation in muscles, normal serum CK levels is a pathognomonic and suggestive laboratory finding of PFMS.^15^ Similarly, even in attack-periods, muscle biopsies are expected to be within normal limits, as shown in our case.^15^

The genetic background of PFMS has been also studied and like overall FMF population, homozygous or heterozygous M694V mutation is the most frequent mutation detected in patients, followed with other common exon 10 mutations including M680I, M694I and V726A (4, 16). As in our case, especially in the absence of typical FMF attack pattern and/or exon 10 MEFV mutations, differential diagnosis of periodic fever syndromes and other auto-inflammatory conditions is also crucial. Although, Turkey is one of the endemic regions for FMF, the prevalence of TRAPS, HIDS and other periodic fever syndromes is not infrequent. However, the absence of long-term fever and ocular findings, mainly periorbital oedema for TRAPS, and absence of lymphadenopathies and gastrointestinal symptoms for HIDS helped to exclude them.

On the other hand, as we found in our case, an exon 3 mutation P369S has been also associated with FMF previously but in current knowledge it is not accepted as a pathologic MEFV mutation, but it contributes an IL-1 beta related mixed auto-inflammatory condition and attributed as in FMF or FMF-like clinical picture in literature.^17,18^ This might be due to the function of exon 3 which encodes B-box zinc finger of pyrin protein that is very important part to regulate interactions of pyrin protein and activate IL-1beta.^19^ The presence of P369S reveals highly different phenotypic features and infrequently associated with typical FMF. However, manifestations usually resemble PAPA or FMF components and IL-1beta activation is the main part of the pathogenesis. Also, in clinical features of FMF, PFMS is a rarer but relatively pathognomonic manifestation and even in in the absence of typical exon 10 mutation or FMF features, it can be evaluated under the title of FMF components. On the other hand, diagnostic, clinical and treatment-based approach in the presence of P369S is also similar to FMF and some patients with P369S mutations respond well to colchicine and IL-1 blockage, if necessary, and exhibits attacks characterised with IL-1 related hyperinflammatory nature with more prominent malleolar lesions and livedoid vasculopathies (17, 18, 20). However, in literature a few cases of PFMS with P369S or compound heterozygosity of P3669S and R408Q were reported.^21^ With the light of all this knowledge, we classified our patient in FMF spectrum autoinflammatory disease, also and used IL-1beta blockage successfully.

In conclusion, PFMS should be considered even in the absence of apparent FMF attack pattern and in the presence of unexpected severe muscle weakness, especially in areas endemic for FMF and long-lasting myalgia attacks. After a well-organised work-up for differential diagnosis, empirical treatment should be given until genetic analysis is obtained in these types of atypical cases. In this kind of rarer presentation, a wide spectrum of suspicious diagnosis makes the use of anakinra rational as the first choice of drug challenge, owing to various effects of corticosteroids in several different conditions.

## Data Availability

Data available on request from the authors. The data that support the findings of this study are available from the corresponding author upon reasonable request and all authors have access all data.
